# Less Is More? The Impact of Trauma Volume on the Positive Rate of Head Computed Tomography Scans in Head Trauma Patients

**DOI:** 10.1100/2012/340317

**Published:** 2012-06-18

**Authors:** Chao-Wen Chen, Yun-Ting Lou, Chi-Ming Chu, Hsing-Lin Lin, Wei-Che Lee, Ke-Zong Ma, Yuan-Chia Cheng, Liang-Chi Kuo

**Affiliations:** ^1^Division of Traumatology, Department of Surgery, Kaohsiung Medical University Hospital, Kaohsiung Medical University, Kaohsiung 807, Taiwan; ^2^Department of Emergency Medicine, Kaohsiung Medical University Hospital, Kaohsiung Medical University, Kaohsiung 807, Taiwan; ^3^Graduate Institute of Injury Prevention and Control, Taipei Medical University, Taipei 110, Taiwan; ^4^Department of Ophthalmology, E-DA Hospital, I-Shou University, Kaohsiung 110, Taiwan; ^5^School of Public Health, National Defense Medical Center, Taipei 84001, Taiwan; ^6^Department of Healthcare Administration and Medical Informatics, Kaohsiung Medical University, Kaohsiung 807, Taiwan

## Abstract

*Objective*. Few studies have assessed the impact of trauma volume on the operational efficiency of emergency departments. Herein, we evaluate the association between trauma volume with the positive rate of head computed tomography scans in head trauma patients in a tertiary care hospital. *Methods*. This is a retrospective cohort review involving all head trauma patients presenting to a tertiary care hospital. Trauma census, head trauma patient volume, the number of emergent head CT scans, and the number of positive head CT scans were collected on a monthly basis. Comparison was primarily made between the trauma patient volume and the positive rate of head CT scans. *Results*. 25,549 trauma patients were reviewed. Of these, 5,168 (20.2%) sustained head trauma and 3,336 head CT scans were performed with mean 29.1% positive rate of substantial head injuries. The monthly data were analyzed and a statistically significant correlation between monthly trauma volume and decrease in positive rate of head CT scan was identified (Pearson *r* = −0.51, *P* = 0.02). With introducing different cut-point values of trauma volume, we identified the threshold of trauma census as approximately 4.9 and 8.8% higher than mean monthly trauma volume in discriminating significant decrease of positive rate of head CT scans.

## 1. Background

Compared with the other departments of a hospital, the emergency department (ED) plays a different and unique role in the aspect of consistency. For providing the same level of medical care 24 hours a day and seven days a week, EDs usually maintain a constant amount of human and technical resources throughout the time. On the other hand, the day-to-day burden of visiting patients keeps testing the effectiveness and quality of care in the ED. In our previous study, we demonstrated that the risk of harboring missed injuries among major trauma patients was not influenced by concurrent trauma census in the ED [1]. However, many emergency physicians still believe that the operation efficiency of EDs is affected by visiting patient flow. Some previous studies have demonstrated that treatment delays and unfavorable outcome of visiting patients were closely associated with ED overcrowding [2–4].

In Taiwan, the National Health Insurance Scheme provides a comprehensive benefit package that covers preventive, regular, and emergent medical care services. Since all citizens have equal and convenient access to acute care facilities, ED overcrowding is common. Despite the fact that ED overcrowding has been enthusiastically discussed and presumed to be a key attribute of unfavorable quality of acute care, there are no published studies regarding the potential misuse or overuse of examination tools in such an overload setting. Focusing on a selected group of head trauma patients, we hypothesized that the utilization of head computed tomography (HCT) might be influenced by different levels of ED census regardless of the existence of a standard HCT guideline. Furthermore, we suspected there is a threshold of trauma volume for discriminating the significant decrease of effectiveness of HCT performed at ED.

To minimize unnecessary radiation or expenditure, selective use of HCT in patients with head trauma has been proposed [5, 6]. In our ED, there is an approved guideline to justify the use of head CT scans (Table 1). Each emergent HCT order for trauma patients should meet at least one criterion. The major purposes of our study were (1) to evaluate the association between trauma volume and the positive rate of head CT scans in head trauma patients and (2) to determine the threshold of trauma volume possessing the discriminating ability of decreased effectiveness of emergent HCT for head trauma patients.

## 2. Methods

The retrospective case review was completed at a 1300-bed tertiary care hospital in south Taiwan. Annually, approximately 85,000 patients visit the ED and are managed in three different services (internal medicine, surgical emergency/trauma service, and pediatric emergency medicine) in accordance with their chief complaints. With the approval of the hospital Institutional Review Board, all head trauma patients admitted to the Emergency Department under the care of the trauma service between January 1, 2007, and December 31, 2008, were entered for analysis. Demographic data, ED treatments, and the radiologic reports focusing on HCTs were obtained by medical record review.

In our study, head trauma refers to any overt damage to the scalp, skull, or brain regardless of mechanism or severity. We identified these head trauma patients based on diagnosis at ED discharge. Superficial dermal abrasions of the head were excluded due to their mild severity with limited impact on clinical outcome. A positive HCT included findings of subdural, epidural, or parenchymal hematoma, subarachnoid hemorrhage, cerebral contusion, or skull fracture. Prominent brain edema was also defined as positive finding if it was documented in the formal radiology reports. Two record abstractors retrieved all key information from medical records after receiving four hours of training for understanding key areas of the abstraction form. The entire process was supervised by a principal investigator (CWC).

The ED database permits calculation of the trauma volume and the number of head trauma patients on a monthly basis. All these data were tabulated as well as the positive rate of HCTs in head trauma patients. For the consistency of performance of trauma service, the variation of the physician staff caring for the trauma patients at ED was also taken into consideration. There are six trauma-attending surgeons equally sharing the work shifts of trauma care in the ED. They supervise and direct all trauma surveys as well as resuscitative procedures in trauma bays. Any month involved in staff change, which might have influenced the quality of care, would be excluded for further data analysis.

Pearson correlation coefficients were primarily computed to examine the relationship between monthly trauma volume and positive rate of HCTs in head trauma patients. By introducing various cut-point values of trauma volume, the monthly census data were then dichotomized as high-volume or low-volume groups. The independent *t*-test was used to compare the differences of positive rates of HCTs from high-volume to low-volume groups. *P* value less than 0.05 indicated a statistically significant difference. On the basis of different definitions of high- or low-volume settings, an ROC curve was also constructed for balancing the sensitivity and specificity as well as to determine the best cut-off value of positive rate of HCT for each setting. The best cut-off value was determined, while balancing the best sensitivity with the lowest false-positive rate. The data were analyzed with SPSS, version 15.0 (SPSS. Inc. Chicago, IL).

## 3. Results

**Figure 1:**
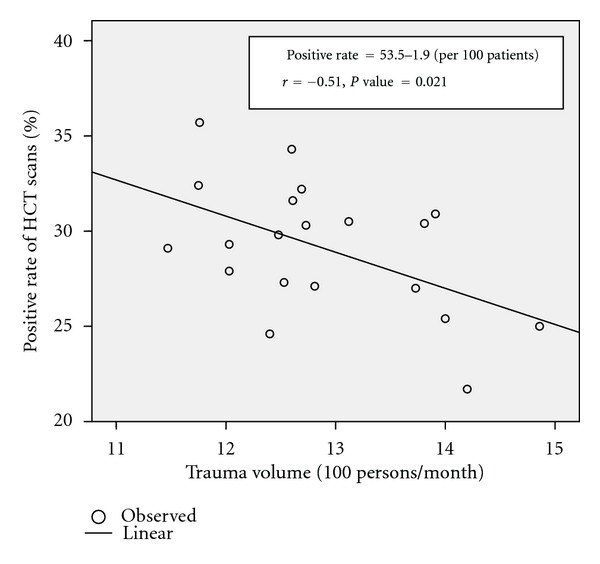
The relationship between monthly trauma volume and positive rate of head CT scans in 20 eligible samples.

During the 2-year period, four nonconsecutive months involving staff changes were excluded. Of 20 eligible months, 25,549 trauma patients presented to the emergency department of the hospital. 5,168 (20.2%) sustained head trauma and 3,336 head CT scans were performed among these at ED. The characteristic data regarding trauma volume, head trauma patients, and HCTs performed in head trauma patients are shown in Table 2. The mean monthly trauma volume and head trauma census was 1287.6 ± 92.7 and 258.5 ± 26.6, respectively. The mean monthly proportion of head trauma patients among all trauma patients was 20.1 ± 1.6%. Head CT scan was performed in over half of all head trauma patients regardless of injury severity (mean 65.2 ± 9.9%). With review of formal radiology reports, the mean positive rate of head CT scans was 29.1% (range, 21.7–35.7%). We conducted a Pearson correlation analysis between monthly trauma volume, rate of monthly head trauma census, and rate of HCTs performed in head trauma patients. No significant correlation was recognized except that a moderate negative correlation between monthly trauma volume and positive rate of HCTs was found (*r* = −0.51, *P* = 0.021) (Figure 1). The summary of the correlations is depicted in Table 2.

**Figure 2:**
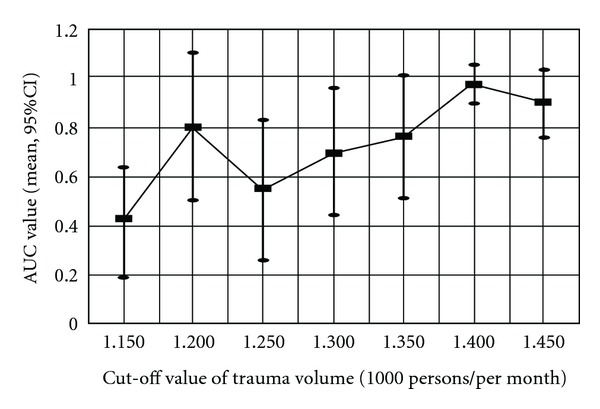
Graph illustrates AUC values for different cut-off values of trauma volume.

For detecting the threshold of trauma volume yielding significant decrease in effectiveness of HCTs, we tested the monthly positive rate with different cut-point values of trauma census by means of independent *t*-test (Table 3). Based on the historical data of monthly trauma volume (mean: 1287.6 ± 92.7; range: 1147–1486), by means of gradually increasing 50 persons/month from 1100 to 1500 persons/month which were recommended by the expert panel of the present study, we set different cut-point values to define whether a monthly trauma volume was of high volume or low volume. When the threshold of monthly trauma volume reached 1,350 (equal to a daily volume of 45 patients) and 1400 (equal to a daily volume of 46.7 patients), the difference between positive rates of HCTs in high-volume and low-volume groups yielded a significant difference. ROC analysis for different cut-off value settings was also performed. These results are integrated into Table 3 and Figure 2. These ROC curves revealed that the best result was obtained using a cut-off value of positive rate of HCT of 0.25 with introduction of a threshold of 1400 persons/per month for defining monthly trauma census as high or low volume (AUC = 0.972, *P* = 0.032).

## 4. Discussion

Our findings demonstrated that trauma volume bears an inverse relationship to the effectiveness of HCTs. The decrease of effectiveness of examination tools in ED has been rarely discussed before. Most previous efforts highlighted the relationship with patient outcome. Theoretically, a threshold of patient volume may exist beyond which the quality of care will be impaired. Increased medical staff workload has been associated with unfavorable outcome in several studies [7, 8]. Limited but diversified quality measures in emergency medicine pertaining to special diseases such as pneumonia, acute myocardial infarction, or asthma as well as the operation efficiency related to special procedures have been proposed [9, 10]. Nevertheless, the perception of quality is inevitably subjective and difficult to be defined. A universally accepted standard measure is yet to be established.

Notwithstanding previous studies regarding quality care in overloaded EDs presenting an inverse relationship between increased ED volume and patient outcomes of some diseases, little is known about the change of cost-effectiveness of examination tools in such messy settings. Because EDs care for different “customers,” it is reasonable to select a specific group of clients to conduct a cost-effectiveness survey. In our study, we selected head trauma patients as study objectives. The major reason is that there are relatively standard strategies to deal with patients sustaining head injuries as well as approved guidelines for utilization of emergent HCT in our institution. The hypothesis of our study is that the standard strategies to approach head trauma patients might be altered in a crowded ED intentionally or unintentionally. Although HCTs are usually performed in moderate or severe head trauma patients, the selective use in minor head trauma patients is still in debate [5, 6, 11]. At our ED, minor head trauma patients might be managed conservatively with treatment including several hours of clinical observation, inpatient care, or just ED discharge with the arrangement of trauma clinic appointments for followup. If patient presentation fulfills at least one criterion of the guideline for emergent HCT, an instant scan would be performed. In our study, the negative correlation between monthly trauma volume and positive rate of HCTs implied that the decision making according to a standard guideline was somehow altered by different levels of patient load. With the increase of trauma volume, the effectiveness of HCTs indeed decreased. The increased workload might cause physicians' decision errors or induce physicians to frequently seek aid from radiologists by way of arranging emergent HCTs, or force them to clear the crowded “battlefield” by means of aggressively discharging patients with negative HCTs. A variety of misuse or overuse of examinations may be related to it. To discuss the adverse effects of ED overcrowding, the decline of effectiveness of examinations warrants more concern.

Positive relationships between hospital volume and outcomes have been demonstrated for some specific surgeries and medical situations [12–15]. Nevertheless, previous research assessing the impact of trauma volume and outcomes has been inconsistent [16–19]. Ideally, centralization of trauma patient to the designated tertiary care hospitals can reach the aim of increasing exposure to trauma cases and enhance specialist training. Realistically, in the dynamic process of emergency care, trauma center is subject to the detrimental effects of high temporal volume. Marcin and Romano found that higher volume than the monthly average census was associated with higher odds of readmission among elderly trauma patients [18].

Due to unmeasured patient and environment complexity, it is difficult to identify a well-defined volume threshold to detect the decrease of quality care in EDs. By means of stepwise increase in trauma census based on historical data, we have demonstrated that the unrefined threshold of trauma volume involving significant decrease of effectiveness of HCTs could be estimated. Focusing on other specific medical or surgical situations, the threshold of census may differ. Hwang et al. found that an ED census greater than 120% of bed capacity significantly impaired the pain assessment with documentation [20]. In another study conducted by Polevoi and colleagues, ED capacity higher than 100% was found to be associated with patients who left without being seen and was most significant at 140% capacity [21]. Similar results were proposed by Hobbs et al. [22]. Our significant result was generated from monthly data, and the value of threshold is approximately 5 percent (4.9% to be exact) higher than the mean monthly trauma volume. It highlighted the operation efficiency of ED staff based on the historical patient flow, instead of ED capacity based on a fixed number of registered beds. On the other hand, with a fixed trauma volume below the cut-point threshold, the significant decrease in positive rate of HCTs may be selected as a quality indicator of a malfunctioning ED. However, further detailed studies relating to daily or even hourly volume might be able to clarify the real impact in a dynamic process.

Limitations of our study arise mostly from the fact that it is a retrospective study of a single institution. The generalizability was restrained and the result of our study cannot be adapted to different populations pertaining to other specific conditions. Second,we did not enroll more data for analysis as there was a duty remodeling process caused by a shortage of ED staff commencing in the beginning of 2009. That process involving permanent staff and duty change blocked our further study. Third, some may argue that the proportion of head trauma patients with different severities would affect the positive rate of HCTs, since the positive rate of HCTs might be lower in mild head trauma patients than moderate or severe head trauma groups. However, since the emergent head CT guidelines regarding all head trauma patients have been established, we merely wished to highlight the ED physicians' decisions of utilizing HCTs for all head trauma patients. Furthermore, according to our electronic medical records, the patients with mild traumatic brain injury almost accounted for one-fourth of all patients during these study periods without overt proportional change. The results of our study simply imply that the decisions seemed less precise in relatively overloaded settings regardless of head injury severity. A sensible decrease of effectiveness of examinations may precede or coincide with the occurrence of medical errors. 

Although our study supports the inference of decreased HCT effectiveness in relatively crowded situations, this still leaves the crucial question unanswered: can we afford the cost of medical errors related to delayed recognition of patients with substantial intracranial lesions in crowded EDs? Future efforts should attempt to outline the undetected relations between ED operational effectiveness and overall outcome.

## 5. Conclusions

Our data showed that effectiveness of emergent HCT decreased in relatively crowded settings. An early warning system forecasting ED overload based on historical operational efficiency different from conventional ED capacity may be developed on the basis of our findings.

## Figures and Tables

**Table 1:** Guidelines for emergent head CT scans for trauma patients. The guideline is modified from “CT rules for mild brain injuries” advocated by the Taiwan Neurosurgical Society http://www.neurosurgery.org.tw/nsr/tbi/main.htm.

Urgent scan is indicated
Moderate and severe head trauma
GCS < 15 two hours after injury
Suspected skull or skull base fracture
Severe craniofacial trauma
Focal neurological deficit
Seizure attack after injury

Optional use of head CT scans
≧2 episode of vomiting
Aged ≧65 years or ≦ 2 years
Drug or ethanol intoxication
Persistent diffuse headache
More than 30 minutes of amnesia or loss of consciousness
since injury
Dangerous mechanism of injury
Road traffic accident—as pedestrian
Road traffic accident—ejected from car
Fall > 1 m or > 5 stairs
Coagulopathy or on anticoagulants

**Table 2:** Characteristic data of trauma patients on a monthly basis (*n* = 20). Univariate correlation analysis showed an inverse weak correlation between trauma volume and positive rate of head CT scans. There was no significant correlation noted between trauma volume and the other two proportions.

Variable^∗^	Median	Mean (SD)	Range	Correlation with trauma volume
Trauma volume (persons/month)	1265	1287.6 (92.7)	1147–1486	—
Rate of head trauma patient (%)	19.9	20.1 (1.6)	16.7–23.2	− 0.03
Rate of HCT scans performed in head trauma patients (%)	64.3	65.2 (9.9)	48.1–89.7	− 0.32
Positive rate of HCT scans (%)	29.6	29.1 (3.4)	21.7–35.7	−0.51**

^∗^All variables showed parametric distribution by the Shapiro-Wilk test.

^∗∗^Pearson's correlation with a *P* value less than 0.05.

**Table 3:** Correlation between monthly trauma volume and other survey variables.

Cut-off value of trauma volume (person/month)	Low volume		High volume				
	Number of months	Positive rate of HCTs	Number of months	Positive rate of HCTs	Overall performance		
	*n*	mean (SD)	*n*	mean (SD)	*P* value^∗^	AUC	*P* value
1100	0	—	20	29.13 (3.44)	—	—	—
1150	1	29.1	19	29.13 (3.53)	0.994	0.421	0.795
1200	3	32.4 (3.3)	17	28.55 (3.21)	0.072	0.804	0.101
1250	7	29.83 (3.49)	13	28.74 (3.49)	0.517	0.549	0.721
1300	13	30.12 (3.09)	7	27.27 (3.49)	0.076	0.703	0.143
1350	14	30.15 (2.97)	6	26.73 (3.49)	0.038	0.762	0.070
1400	18	30.02 (2.79)	2	24.03 (2.03)	0.002	0.972	0.032
1450	19	29.34 (3.39)	1	25	0.228	0.895	0.193
1500	20	29.13 (3.44)	0	—	—	—	—

HCT: head computed tomography.

^∗^Correlation is significant at the 0.05 level (2-tailed).
